# Single-Cell Multiomics Analysis for Drug Discovery

**DOI:** 10.3390/metabo11110729

**Published:** 2021-10-25

**Authors:** Sam F. Nassar, Khadir Raddassi, Terence Wu

**Affiliations:** 1 Department of Biology, Brandeis University, Waltham, MA 02453, USA; 2 IsoPlexis, Branford, CT 06405, USA; 3 Department of Neurology, Yale School of Medicine, New Haven, CT 06511, USA; khadir.raddassi@yale.edu; 4 West Campus Analytical Core, Yale University, West Haven, CT 06516, USA; terence.wu@yale.edu

**Keywords:** multiomics, genomics, metabolomics, proteomics, transcriptomics, single-cell, mass spectrometry, IsoLight, COVID-19

## Abstract

Given the heterogeneity seen in cell populations within biological systems, analysis of single cells is necessary for studying mechanisms that cannot be identified on a bulk population level. There are significant variations in the biological and physiological function of cell populations due to the functional differences within, as well as between, single species as a result of the specific proteome, transcriptome, and metabolome that are unique to each individual cell. Single-cell analysis proves crucial in providing a comprehensive understanding of the biological and physiological properties underlying human health and disease. Omics technologies can help to examine proteins (proteomics), RNA molecules (transcriptomics), and the chemical processes involving metabolites (metabolomics) in cells, in addition to genomes. In this review, we discuss the value of multiomics in drug discovery and the importance of single-cell multiomics measurements. We will provide examples of the benefits of applying single-cell omics technologies in drug discovery and development. Moreover, we intend to show how multiomics offers the opportunity to understand the detailed events which produce or prevent disease, and ways in which the separate omics disciplines complement each other to build a broader, deeper knowledge base.

## 1. Background

Determining the proper combination of properties such as activity, toxicity, and exposure is a complex process, and one of the keys steps in the design of drug candidates and their subsequent advancement to the development stage. Dubbed as ‘the rule of three’, the activity–exposure–toxicity relationship demonstrates the most difficult challenge in conducting successful drug design and development [1,2]. The drug discovery process starts with the identification of potential targets for drug candidates’ effects. In order to be considered acceptable, a drug must be efficacious, safe, and meet the applicable clinical and commercial needs. However, due to various circumstances, formulating an unambiguous assessment of these measures can be challenging, particularly when considering how to appropriately define the concept of drug target efficacy [3,4,5]. Regarding activity, a drug needs to first bind to its target, and subsequently impact the function of this target, in order to have a significant effect; such targets may include specific genes, proteins, or other biomolecules. Therefore, the efficacy of a target should be evaluated by its potential in delivering effective therapeutic treatments. Regarding exposure, the active moiety (drug or metabolite[s]) enters the systemic circulation, thereby accessing the site of action. Drugs given intravenously will have an absolute bioavailability of 100%, while drugs given through different routes typically possess an absolute bioavailability of less than 100%. Even with a bioavailability of 100%, many drugs do not actually hit their intended molecular targets. Genetic variations can produce underlying racial, gender, ethnic, and population differences in drug disposition and response. Regarding toxicity, the objective of safety evaluation is to determine any inauspicious consequences associated with target modulation. Unavoidable on-target toxicities and clinical adversities constitute potential safety challenges during the process of drug target identification and prioritization [6,7,8].

## 2. Introduction of Omics in Drug Discovery

Omics approaches provide useful tools for characterizing and quantifying pools of biological molecules and exploring their functions, relationships, and actions in the cells of living creatures. High-throughput technologies are playing an ever-more important role in the everyday methodology of biological researchers, and have unlocked exciting new areas for medical research. Multiomics are separate, distinct disciplines, yet have become increasingly intertwined with others to advance strategies for diagnosing diseases, evaluating treatment responses, and other techniques in the burgeoning field of personalized medicine. Upon the original discovery of a drug target, our understanding of its full therapeutic potential may be limited; the rapidly emerging field of omics studies shows tremendous potential to deepen and broaden this vital knowledge [9,10,11]. As an example, genomics and transcriptomics applications are pushing the development of many new research and clinical disciplines within diagnostics, therapeutics, and the pharmaceutical industry. These areas extend from gene therapy applications, pharmacogenomics, and disease prevention to developmental biology, as well as evolutionary and comparative genomics [12,13,14,15]. Given the high throughput of current omics technologies, a wide array of measurements of potential targets, such as DNA, RNA, protein, and metabolites, is driving rapid improvements in the quality, quantity, granularity, and cost of methods for drug discovery research. Discovery, development, and linkage of targets to drug candidates becomes much more thorough and robust through application of genomics, transcriptomics, proteomics, and metabolomics data. It is becoming increasingly apparent that these new technologies, such as proteomics, metabolomics, and lipidomics, are proving indispensable to a fuller understanding of biology down to the phenotype level and beyond. Researchers have come to favor the integration of additional multiomics approaches, as they provide strong support for their efforts to generate multilevel medicine techniques, and expand the capabilities of precision medicine.

The major function of multiomics techniques is the detection of proteins (proteomics), mRNA (transcriptomics), genes (genomics), metabolites (metabolomics), and lipids (lipidomics) in a specific biological sample. These methodologies, working together or individually, and the data they produce, have a broad range of applications. Improvements in microarray technology are fueling strong progress and innovation in genomic and transcriptomic research. Proteomics and metabolomics rely on mass spectrometry as the most frequently used method for the detection of analytes in biological samples. Sorting, understanding, interpreting, and integrating the enormous amounts of data generated by these disciplines requires ever-more skilled bioinformaticians, along with more powerful computing ability. These efforts promote greater specialization of diagnostic tests and treatment responses, leading to more personalized treatment and therapeutic options. Developments in single-cell omics research allow for insightful analysis of the variation among cells and the impact of cellular functions on their environmental responses, down to a molecular scale.

### 2.1. Genomics: The Study of What Can Happen

Genomics is changing the direction of disease diagnosis and treatment, and, in turn, is gaining traction in drug development. The focus is shifting from seeking new chemistry to a better understanding of the underlying biology. The genome of a cell or organism provides its complete sequence of DNA, which is typically located in the cell nucleus or in other organelles, including the chloroplast or mitochondria. With the exception of cases of mutations and chromosomal rearrangements., the genome remains remarkably stable, even over long periods. One recent experimental development is the single nucleotide polymorphism (SNP) chip, which provides thousands of oligonucleotide probes, which bind to particular DNA sequences with known nucleotide variants. DNA sequencing reveals insertions, deletions, and copy number variations (CNVs) in the genetic material. Each of these alterations indicates a deviance from the original parental genes, including modifications produced by methylation of cytosines. A recent and more powerful technology is personal genome sequencing, which produces direct and complete sequencing of genomes and transcriptomes (Table 1) [16]. Genomics techniques are gaining interest in the examination of an individual’s microbiome as it relates to their health and disease [17,18,19,20,21].

Modified RNA is crucial in cell proliferation, migration, invasion, and epithelial–mesenchymal transition. Cancer cells are the final product of a complex mixture of genetic, epigenetic, and RNA alterations, any one or a combination of which play major roles in drug resistance, and present major challenges in disease prognosis and treatment. Recent investigation into the roles of RNA modification has focused on use of such modifications to enhance the targeting ability of several chemotherapeutic drugs, including cisplatin, 5-fluorouridine, and gefitinib [22]. The knockdown of methyltransferase-like 3 (METTL3) in pancreatic cancer greatly improved sensitivity to drugs such as 5-fluorouracil, cisplatin, gemcitabine, and irradiation. These targeted epigenetic alterations offer new avenues to elimination of drug resistance and, in turn, stronger cancer treatments [23].

### 2.2. Transcriptomics: The Study of What Appears to Be Happening

Transcriptomics signals are critical for clinical candidate selection in that they provide an assessment of the potentially harmful impacts of drugs and their interactions with potential targets at early stages of drug development. The transcriptome is the set of all RNA transcripts expressed in a cell or tissue. This includes coding transcripts of messenger RNA (mRNA), transfer RNA (tRNA), ribosomal RNA (rRNA), and microRNA (miRNA), as well as non-coding RNA (ncRNA). However, protein-coding genes of the transcriptome make up just 1.5 to 2 percent of the human genome. Microarrays and RNA sequencing (RNAseq) provide the preeminent technologies for transcriptome measurement. While the conventional microarrays rely on hybridization probes to detect specific RNA transcripts, RNAseq is a novel method that does not require probes for RNA sequencing (Table 1) [16]. The inherent differences in a variety of omics datasets present serious challenges when it comes to quality control, quality assurance, data normalization, and data reduction methods. RNAseq datasets, for instance, comprise tens of thousands of transcripts, whereas small RNAseq datasets may involve numbers in the low thousands or even hundreds; this leads to issues during normalization and scale.

Detection of drug-induced genome-wide gene expression changes can be achieved through RNAseq, for example, by screening a series of biomarkers and candidate drugs associated with age-related macular degeneration. Using RNAseq technology, it was found that nicotinamide can impact the disease by inhibiting drusen protein, inflammation and complement factors, up-regulated nucleosome, ribosome, and chromatin-modifying genes. Based on these findings, the researchers suggested that nicotinamide could be an effective component in developing treatments for age-related macular degeneration [24].

### 2.3. Proteomics: The Study of What Makes It Happen

The proteome constitutes the complete set of proteins from a cell, tissue, or organism at a particular time. There are numerous factors contributing to the inherent complexity of the proteome; proteins may undergo posttranslational modifications to their component amino acids. Such modifications include, but are not limited to, glycosylation, phosphorylation, acetylation, and ubiquitylation. Additionally, there may be variations in intracellular locations and spatial configuration, along with interactions with other proteins and molecules. Both label-based and label-free quantitative approaches involve sample collection and protein extraction, followed by their breakdown into peptides through enzymatic or chemical digestion. Samples then undergo separation/fractionation of the digested protein mixtures with appropriate electrophoretic and/or chromatographic methods. Subsequently, the applicable pathways and networks are analyzed by bioinformatics tools, an additional step to the identification and quantification processes of the mass spectra that provide relevant information at both peptide and protein levels [25]. While earlier methodologies generally involved 2D-PAGE-based (dye/fluorescence labeling) protein spot extraction with subsequent MS characterization through matrix-assisted laser desorption/ionization time-of-flight (MALDI-TOF) or LC-MS, present techniques tend to adopt more streamlined approaches. These labeling techniques include Stable Isotope Labeling with Amino Acids in Cell Culture (SILAC), Tandem Mass Tags (TMT), Isobaric Tagging for Relative and Absolute Quantitation (iTRAQ), 18O Stable Isotope Labeling, and Isotope-Coded Affinity Tagging. These techniques make use of label-based approaches which involve quantitative steps, allowing for greater system-wide screening. On the other hand, label-free methodologies are steadily gaining more interest in the field of proteomics, as they are less expensive, and faster with high throughput, yet equally reproducible and accurate as label-based ones. In particular, these techniques, such as Spectral Counting and LFQ (Label-free quantification), perform protein quantification by counting the number of spectra identified for a given peptide, or measuring precursor signal intensities [26,27].

Proteomics tools are useful in identification of numerous proteins which are altered in the presence of disease. Another important use is for identifying biomarkers from biological fluids during drug discovery. For example, plasma proteomics is reported to identify biomarkers and pathogenesis of COVID-19 [28]. Plasma proteomics techniques served to profile host responses in a group of COVID-19 patients, including non-survivors, as well as those with a range of symptom severity. The study revealed that COVID-19 led to a significant variety of alterations to plasma proteins. One such discovery was a compact biomarker combination containing 4 proteins, including orosomucoid-1/alpha-1-acid glycoprotein-1 (ORM1/AGP1), ORM2, fetuin-B (FETUB), and cholesteryl ester transfer protein (CETP).

### 2.4. Metabolomics: The Study of What Actually Happens

Metabolomics is a powerful tool for cell analysis, with the ability to develop an extensive presentation of the cellular state through its pool of metabolites. This capability makes it an effective method for developing more personalized, precise approaches for diagnosis, monitoring, and evaluation of patients’ treatment response [29]. The metabolome provides a composite view of the entire metabolic activity of the designated sample, which can include a complete organism, organ, tissue, or cell. The metabolic intermediates of a given sample include metabolic intermediates originating from a wide array of biochemical pathways. Among these molecular metabolites are endogenous substances such as nucleic acids, amino acids, carbohydrates, and lipids, along with hormones and other signaling molecules, as well as exogenous substances such as drugs and their metabolites. All of these taken together provide a functional overview of the metabolic status, cellular activity, and global biochemical events associated with a biological system. The metabolome is dynamic; disease, stress, changes in diet, physical activity, and pharmacological effects produce variances both within a single organism, and among those of the same species. Given this depth and breadth of information, the field of metabolomics provides a useful mechanism for conducting drug tests, with the capability of more precisely analyzing differences in phenotype. Metabolites are able to present a functional representation of phenotype, due to their ability to provide direct signatures of biochemical activity, in contrast to genes and proteins, which are defined by regulatory mechanisms including post-translational modifications of proteins and epigenetic regulation [30].

Metabolomics analyses start with experimental design, as approaches differ for targeted or untargeted analyses, and are dependent on the aims of the study. Targeted analyses and hypothesis-generating data are used to validate distinct hypotheses, while untargeted analyses are mainly used in the discovery phase. The study requirements determine next steps, and these vary widely based on which platform is used for sample analysis. The study question, experimental design, and choice of platform each influence suitable sample collection strategies, methods for quenching of metabolism, optimized metabolite extraction and sample reconstitution, optional chemical derivatization, MS (with or without a chromatography interface), or NMR. The final measures comprise the data analysis, with steps for statistical analysis, data alignment, imputation, annotation filtering, and pathway/network analysis. Notably, data structure, imputation methods, metabolite identification, normalization, scaling, and transformation are greatly dependent on the specific instruments and data types.

Metabolomics and SARS-CoV-2: Recent discoveries of several links among the recent SARS-CoV-2 virus, diabetes, and other metabolic disorders, have suggested that immunometabolism plays an important role in infection. One group, using both NMR and LC/MS, compared whole blood metabolite samples from 17 SARS-CoV-2-positive patient samples against 25 SARS-CoV-2-negative healthy controls. Several markers of inflammation were discovered in samples from patients with COVID-19: increased alpha-1-*cis* glycoprotein signal A, an increased kynurenine:tryptophan ratio, and the modulation of several lipid profiles, including high density and low-density lipoproteins, and an increase in triglycerides [31].

### 2.5. Lipodomics: The Study of What Actually Happens

Lipids are essential metabolites that have many crucial cellular functions, and can provide a direct readout of cellular metabolic status. Lipidomics is the study of the lipidome, which represents the total lipid content in a cell. This is expected to be in the range of tens of thousands to hundreds of thousands of molecules, at concentrations between amol/mg and nmol/mg of protein. Lipidomics, when combined with wide use of mass spectrometry instruments, is a rapidly advancing field; for example, the combination is greatly enhancing the development of lipid biomarkers to study disease states. The study of cellular metabolism is broadened through the quantification of changes of individual lipid classes, subclasses, and molecular species that reflect metabolic differences. Having already studied pathways and networks for lipid metabolism, such changes in lipid quantities provide clues to variations in enzyme levels, activities, and/or gene expression patterns. Importantly, this approach enables us to study cellular metabolism by quantifying the changes of individual lipid classes, subclasses, and molecular species that reflect metabolic differences. As the pathways and networks of lipid metabolism have been extensively studied, any changes in lipid amounts can simultaneously reveal variations in several enzymatic levels, activities, and/or gene expression patterns.

As an example of applications of lipidomics in disease biomarker discovery, a study used LC-MS lipid profiles of fasting blood samples from healthy twin pairs, with an average age of 18 years, in conjunction with statistical analysis to study the contribution of genetic background and/or environmental exposure to the blood plasma lipid profile. A total of 54 participants from 23 families were selected for this study. The results demonstrate that similarities in genetic background and/or environmental history among individuals indeed yield similar lipid profiles. Additionally, the authors stated that lysoPCs and SMs distinguish samples from different families better than other lipid classes. This study suggests that useful information on healthy or diseased states can be obtained by monitoring global lipid profiles [32].

## 3. The Importance of Single-Cell Multiomics and Its Impact on Drug Discovery

The heterogeneous nature of a population of cells makes multiomics particularly crucial for conducting single-cell research. Though it was initially presumed that cells of the same lineage held negligible differences, it has been revealed that, even within populations, the phenotype of individual cells may exhibit significant deviations from other cells, and even the population average. The variations in a given cell population are analogous to the differences found among each person in a certain city, state, or country, and how they compare to people in other cities, states, or countries. Identifying and characterizing the biological “noise” that these distinctions represent provides critical information to the study, and poses a substantial challenge for the researcher. At any particular time, differences in timing, pathway, and stimuli response can potentially reveal the history, present state, and imminent future of a specific cell. Various factors, including genetics, cellular history, varied developmental or cell-cycle stages, cell age, and microenvironment, have been shown to engender these variations [33,34,35,36,37,38,39].

Rapid development has enabled single-cell technologies to serve as a valuable tool for analyzing complex cellular networks and characterizing the heterogeneity within cell populations. At the current stage, this has significant implications for disease characterization, among a number of other biomedical fields, and microsystem technologies have shown to be of particular interest for use in engineering sciences. Cancer presents a striking need for single-cell analysis due to the fact that a single irregularity in the body’s 30 trillion cells has the capacity to cause disease. Utilization of single-cell multiomics techniques may enable greater detection of cancer genesis and progression, as well as similar variations of cellular functions which could help produce earlier detection of various medical conditions. Additionally, in order to inhibit drug resistance and develop novel, more effective therapeutics, it is necessary to examine the phenotypic heterogeneity within the same cancer by distinguishing between cells with distinct metabolomes [40,41,42,43,44,45]. Though adjacent cells in an organism emanate from identical genomic and morphological information, individual cells have drastic differences in gene expression, and consequently their metabolite levels and dynamics, due to the divergent circumstances or epigenetic variations that impact each cell. In spite of the potential offered by recent technologies, conducting effective single-cell analysis remains difficult due to the small size of single cells. Metabolite analysis poses a noteworthy challenge because, unlike genes, it is not possible for metabolites to be amplified. On that account, a myriad of techniques has been developed and tested to better examine the small quantity of metabolites present in a single cell. Advancements in both detection sensitivity and ionization techniques, particularly those utilizing mass spectrometry, have begun to prove effective in providing answers to this challenge.

## 4. Single-Cell Technologies

### 4.1. Flow Cytometry

One of the tried-and-true methods for characterizing, analyzing, and isolating single cells based on protein markers is flow cytometry. Historically, researchers have discovered characteristic cell-surface biomarkers and intracellular biomarkers to distinguish certain types of cells, and their differentiation into specialized cell types. Flow cytometry uses antibodies conjugated to fluorophores for detecting cells, which express these specific, distinguishing biomarkers. This method is high-throughput, and can be used to isolate live cells through fluorescence-activated cell sorting, or FACS™. Based on the detection of a fluorescent signal, cells can be deposited singly or in bulk, making this method amenable to the use of downstream single-cell sequencing techniques. Use of FACS™ in single-cell research is widespread, and with good reason. At a cost of approximately $10 per sample, flow cytometry can provide a detection limit of 100–300 molecules/single cell, throughput is about 40,000 cells/s, with analytical efficiency of 90%. One issue with flow cytometry is that even the best sorting methods still cannot accurately identify the time dependent changes that are necessary to define the complete metabolome of the sample [46,47,48].

### 4.2. Mass Spectrometry (MS)

Mass spectrometry shows remarkable potential as a method for conducting potent single-cell analysis. Utilizing MS technologies is particularly useful for single-cell metabolomics with the capacity to examine cell subpopulations, and even make advances when metabolic phenotypes are known. For instance, differences in gene regulation and metabolite levels were observed across the cell lives in a population-level study of cell-cycle-synchronized yeast cells. This demonstrates that the age of parent cells directly influences the phenotypes of clonal cells, and highlights the impact of synchronization and sorting on the metabolome. In addition, a number of mass spectrometric techniques, such as laser-based mass spectrometry, inductively coupled plasma mass spectrometry (ICP-MS), electrospray ionization mass spectrometry (ESI-MS), and secondary ion mass spectrometry (SIMS), have proved useful in elucidating the molecular profiles of individual cells. Single-cell proteomics tools, with recent developments in sample processing, separations, and MS instrumentation, now have the capacity to quantify >1000 proteins expressed within a single mammalian cell [49,50,51,52,53,54,55]. By comparison, just a few years ago, this degree of analysis would have necessitated the input of thousands of cells. This capacity to examine biological systems at such a granular level provides a significant advancement to biomedical research. The biggest technical challenges still facing these different single-cell omics approaches by mass spectrometry are sensitivity, cost, low throughput, long sample preparation and run times, and poor recovery rates.

### 4.3. Mass Cytometry

Another method of protein profiling, called mass cytometry (CyTOF), incorporates aspects of both flow cytometry and mass spectrometry (MS). The basis of this method is similar to flow cytometry, though instead of using fluorescent dye-conjugated antibodies, they are conjugated to heavy metal ions, allowing antibody binding to be detected with the precision of mass spectrometry. Accordingly, the type of instrument that is used is contingent on the experimental targets and objective of the study. As CyTOF techniques continue to develop, and additional isotopes are made available, the capacity to detect parameters will grow, with 45 parameters being accessible as of now. These capabilities allow researchers to generate high-resolution phenotypic and functional profiles of cells in both normal and diseased states, which was unfeasible previously [56,57,58,59,60].

### 4.4. IsoLight

This is a single-cell chamber equipped with multiplex ELISA cytokine capture. Each immune cell is different; thus, the complete characterization of each individual cell is critical. While transcriptome is measured via RNAseq, and surface phenotype is measured via CyTOF or flow cytometry, the overall cellular definition is incomplete without measuring extracellular cytokines that are doing the work in tumor immunology. IsoLight technology can directly detect the functional cytokines of each immune cell. This technology captures the true single-cell protein secretion, with the ability to monitor over 30 biomarkers, providing a full range of function that allows researchers to phenotype each immune cell by its extracellular function. This breakthrough technical innovation allows for complete single-cell functional characterization, something that was not possible previously. The automation of this process saves time and resources, allowing researchers to gain insights into their samples much faster, compared to status quo and bead-based technologies. Single-cell functional proteomics uniquely reveal correlates that lead to biomarker breakthroughs by detecting what each immune cell secretes in a highly multiplexed manner [61,62].

Secreted proteins, and in particular cytokines, have been shown to be key mediators of intercellular communication within the immune system in response to cancer, infectious agents, neurodegenerative disease, traumatic injury, and autoimmune disorders. As a result, determining the quality of single immune cell polyfunctional response, or functional phenotyping, is necessary for understanding positive patient outcomes to immune-mediated therapies, a far better correlate than traditional bulk analysis [63,64,65,66]. Currently, technologies used in single-cell immune analysis for measuring multiple secreted proteins per cell have been reported [67,68,69,70]. IsoPlexis’ platform provides the ability to capture a greater range and variety of functional proteins (cytokines, chemokines, growth factors, etc.) at the single-cell level. This allows for the detection of rare highly polyfunctional cell subsets that are orchestrators of response against tumors or pathogens, and are highly correlative of response, potency, and persistence in various studies across different clinical settings [44,62,71,72,73,74,75,76,77,78,79].

## 5. Applications of Single-Cell Omics in Drug Discovery and Development

A single-cell multiomics approach is the ideal way to achieve full resolution, revealing the complex interplay between genotype and phenotype. Single-cell analysis techniques allow researchers to examine the cell-to-cell variation present in organs, tissues, and cell cultures. The ability to analyze a population of cells at a granular level makes it possible to analyze specific biological mechanisms and systems, and has the potential to generate significant advancements in many areas of biomedical research. In the past decade, a greater understanding of cellular heterogeneity has prompted the rapid development of numerous single-cell omics tools, such as highly multiplexed mass cytometry and microchip-based platforms. Fluorescence flow cytometry (FFC), for instance, remains the most widely used method for protein analysis in single cells. An alternative to flow cytometry is mass cytometry, or CyTOF, which utilizes rare metal isotopes in place of the traditional fluorochrome tags for labeling antibodies, which allows more than 50 proteins to be assayed from a single sample. Both methods involve a similar course of action for measuring secreted cytokines; blocking protein secretion is the first step, after which the cells are fixed and made permeable to enable perfusion of the dye-labeled antibodies. The process of blocking cytokine secretion results in a severe perturbation to cellular function; this may, in turn, affect the accuracy of functional measurements of cellular secretion. In spite of this difficulty, single-cell analysis offers the potential to provide insight into the biological heterogeneity that is present, giving it a significant role in the diagnosis and treatment of diseases.

In drug discovery, changes in the cell function or immunophenotype of drug candidates can be screened with single-cell omics, using ex vivo or in vivo designs. In the preclinical stage, omics technologies are valuable for evaluating the immunophenotype of cell populations in matrices, including whole blood and tissue, to evaluate the immunotoxicology of potential drug candidates. Moreover, single-cell omics can be used to assess specific pharmacodynamic (PD) markers for further elucidation of on- or off-target effects. It is also useful in the clinical stage for exploring the effects of toxicity and making safety, receptor occupancy, and PD assessments. As single-cell omics analysis becomes more widely used in basic research, the appreciation for the heterogeneity of cells in biological systems will continue to grow, leading to the potential for development of new diagnostics and treatments for a number of disease states. Single-cell analysis, from multiomics, can help researchers to advance their understanding of cell heterogeneity by offering the right research tools for a broad range of experimental questions. To conduct effective drug development, it is necessary to augment the speed of the process, and act cost-effectively. Integration of an array of techniques to streamline research and development (R&D), and utilizing genetic data are the best methods to determine the best drug candidates. The fields of proteomics and transcriptomics provide the foremost capabilities for this. The development of probe-based MS for biological molecular imaging has led to a wealth of knowledge and insight into the spatial distribution of various molecules, for a better understanding of physiological processes [80,81].

### Several Examples Are Described Below

A multiomics study seeking to characterize the immune response in COVID-19 has been reported, as shown in the Supplementary Materials Figure S1 [82]. This study used bulk proteome and metabolome measurements, along with single-cell transcriptomic and proteomic assays in 265 samples from 139 COVID-19 patients, as well as samples from 268 healthy subjects. The researchers sought to investigate the immune response to SARS-CoV-2 infection, and how it relates to disease progression and severity (Figure S1). It was discovered that patients’ immune profiles showed more significant differences between those with mild and moderate disease, and the profiles of those with moderate to severe COVID-19. This data allowed the team to build a “severity-dependent, cross-omics correlation network” which revealed links to various omics data and the severity of subjects’ COVID. They realized that elevated inflammation levels, combined with lipid depletion in plasma, were correlated with disease severity. This shift was more dramatic between mild and moderate cases, while proteomics profiles were much more similar between moderate and severe cases. The team concluded that the disparity was induced “by the preferential loss of lipids, amino acids, and xenobiotic metabolism, and significant elevation of inflammatory cytokines” [82].

Another study reported the use of single-cell lipidomics with secondary ion mass spectrometry for characterization and imaging of individual *Aplysia californica* neurons. Further work with tandem mass spectrometry (MS/MS) revealed a major component of the neural membrane, 1-hexadecyl-2-octadecenoyl-sn-glycero-3-phosphocholine [PC(16:0e/18:1)] [83].

In a study involving plasma, a comparison of the arginine:kynurenine ratios successfully distinguished COVID-19 positive samples from those of healthy controls. Plasma samples from suspected COVID-19 cases (ICU admitted) underwent measurements of 183 metabolites, using direct injection LC-MS/MS and ^1^H-NMR approaches. Groups of confirmed (COVID-19^+^) and negative (COVID-19^−^) cases were identified, while another cohort of healthy individuals, matched for age and sex, served as controls. The profile for the COVID-19^+^ group proved unique, with altered levels of kynurenine, creatinine, arginine, sarcosine, and lysophosphatidylcholines. Given that tryptophan degradation is the first step on the kynurenine pathway, and leads to energy generation in the form of NAD^+^, rising kynurenine levels indicate increased tryptophan degradation. This degradation is a product of the release of interferon-γ from activated T cells; meanwhile, reduced arginine may be attributable to its role in tissue repair. Another study involving COVID-19 patients compared blood samples from 102 individuals with COVID-19 (both ICU and non-ICU-admitted) with 26 non-COVID-19 patients (including ICU and non-ICU-admitted). Several omics databases were integrated, among them metabolomics via GC-MS and AEX LC-MS/MS, proteomics via NanoLC/MS/MS, lipidomics via LC/MS, and transcriptomics via RNAseq. It was found that triglycerides were elevated, while other metabolites linked to decreased inflammation, such as citrate, were reduced [84].

This study generated a vast quantity of omics data, at both bulk and single-cell levels. Patients’ plasma was analyzed for a total of 464 proteins, using Olink Proteomics proximity extension assays. The metabolomics firm Metabolon provided mass spectrometry-based measurements of 1,050 metabolites in the patients’ plasma. For single-cell analysis, IsoPlexis’ cytokine profiling assay measured cytokine production in peripheral blood mononuclear cells. Collection of transcriptomic, surface protein level, and TCR and BCR sequence information was achieved using 10X Genomics’ Chromium Single-Cell Kits. Subjects’ blood was drawn twice, once soon after initial diagnosis of COVID, and the second several days afterward [85].

## 6. Conclusions

Multiomics approaches such as genomics, transcriptomics, proteomics, and metabolomics analyze biological samples; each type of analysis can generate and add important information. All aspects of omics are complementary, e.g., proteomics reveal biological function, while genomics or transcriptomics cannot. Streamlined acquisition of genomics and transcriptomics data requires the ability to easily amplify nucleic acid, followed by the use of sequence identity to provide reliable quantification and molecule annotation. Analysis of all genetic material in a biological sample, such as DNA and RNA, is achievable with current sample preparation protocols. Interpretation of this data within biological function is limited by an incomplete understanding of the impact of certain variants on phenotypic variation. Another difficulty is the lack of protein amplification techniques, which require larger sample sizes; additional obstacles revolve around membrane protein isolation, protein insolubility, and problems in detecting small protein quantities. Amplification is also an issue with metabolite testing. The value of metabolite data is further reduced by the fact that less than 30% of the full mass spectra may be identified and quantified. Additionally, score-based spectral annotation of molecules leads to problems with false positives. Given the wide range of sample handling techniques and experimental platforms, the chemical heterogeneity of small molecules, differences in quantification techniques, unstandardized data formats, and analysis pipelines, it is clear there are many challenges to be overcome in gaining all the benefits the full array of omics disciplines have to offer.

Single-cell technologies are playing an important role in the research of drug discovery and development at the molecular level. Potentially, single-cell research can delve into cellular metabolic function, and give us invaluable insights into the most fundamental biological units. The combination of single-cell omics studies has the potential to answer many difficult questions arising from stand-alone disciplines. These new techniques have the ability to simultaneously analyze genomics, transcriptomics, metabolomics, and/or proteomics, in a single cell with one workflow. As such, they should provide an incredible amount of data to uncover novel biological processes, gene-regulatory mechanisms, and cell subpopulations. This knowledge will help to reveal links between cell genotype and phenotype, and its high resolution will improve tracking of individual cell lineages. Herein, we have provided examples of the benefits of applications of single-cell omics technologies in drug discovery and development. Moreover, we have shown how multiomics offers the opportunity to reach far deeper into the detailed mechanisms which produce or prevent disease.

Despite the rapid advancements that have been made, and the remarkable promise that single-cell analysis offers, there are still a number of challenges to overcome. Issues concerning sample considerations, isolation of single cells, and further data analysis continue to present considerable limitations. One issue here arises from the need to quantify the metabolites, as it is difficult to synthesize thousands of possible or actual metabolites. Finally, we must be able to track and understand the intermediate metabolites. For the various single-cell proteomics approaches by mass spectrometry, the most significant technical challenges that remain include sensitivity, cost, low throughput, long sample preparation and run times, and poor recovery rates. Improvement to the usefulness of mass spectrometry will require the replacement of liquid chromatography with gas phase, and the reduction of separation time to seconds, along with independent and streamlined data acquisition; these changes will result in detecting more peptides.

Robust experimental design and execution are the keys to the successful application of an integrated omics workflow. Optimization of sample collection and preparation, along with the capacity for single-step analysis of biological materials, must be the goal for advancing experimental protocols. The ability to produce multiple omics datasets in a single run improves comparability, as well as reducing batch effects and technical variations, which can prove difficult when using high-dimensional data generation processes.

## Figures and Tables

**Table 1:** Omics applications used in pharmaceutical research and development.

Key Point	Genomics	Transcriptomics	Proteomics	Metabolomics
Definition	A sub-discipline of genetics that concerns the sequencing and analysis of an organism’s genome, with a focus on the structure, function, evolution, mapping, and editing of genomes.	The study of the total RNA or mRNA present in a cell or tissue.	Analysis of the entire protein complement of a cell, tissue, or organism within a specific set of parameters.	Large-scale study of small molecules, commonly known as metabolites, within cells, bio-fluids, tissues, or organisms; the metabolome refers to their interactions within a biological system.
Sample	Genomic DNA and RNAs from all types of tissues.	mRNAs from all types of tissues.	All types of tissues and bio-fluids; most commonly used fluid is plasma.	Bio-fluids, such as urine or plasma; tissue extract; in vitro cultures and supernatants.
Techniques	(a) Sequencing of DNA segments that contain methylated fragments after DNA modification with sodium bisulfate or (b) Genotyping using genome-wide oligonucleotide arrays.	DNA-array for quantifying expressed genes (through mRNA levels) Another valuable tool is RNASeq, which aids in studying gene expression and identifying new RNA species.	Identification of peptides/proteins can be determined using MS/MS based strategies. MS utilizes separation techniques including gels (i.e., 2DE), chromatography, and numerous enrichment methods (e.g., antibodies, protein-tags)	The most widely used analytical tools for metabolomic studies to identify large numbers of metabolites are proton NMR (1H-NMR) spectroscopy, GC-MS, and LC-MS. Hundreds of metabolites can be separated and measured in samples of interest such as plasma, CSF, urine, or cell extracts using a diversity of commonly used metabolomics tools, such as NMR, GC-MS and LC-MS detection.
Data processing	Bioinformatics methods (such as Annovar; Circos; DNAnexus; Galaxy; Genome Quest; Ingenuity Variant Analysis; VAAST) are used to detect association of gene(s) with disease, and genome analysis involved hierarchical clustering.	Clustering is used to identify the gene sets; then data analysis is used for gene interpretation. This method can integrate microarray data with prior knowledge on the implication of genes in biological processes (Gene spring; Feature extraction; R; Oncomine; Ingenuity Pathway Analysis, Hierarchical, DAVID Bioinformatics Resources; Panther).	Protein identification and analysis are performed by a variety of bioinformatics tools (such as Mascot; Progenesis; MaxQuant; Proteios; PEAKS CMD; PEAKS Studio; OpenMS; Predict Protein; Rosetta), which are available to researchers. Measurement (random) and systematic (bias) errors are necessary components of proteomic analysis.	To generate and interpret the metabolic profile of the sample, data generated are combined with multivariate data analysis such as partial least square, clustering, discriminant analyses (examples of metabolomic software; BioCyc –Omics Viewer; iPath; KaPPA-View; KEGG; MapMan; MetPa; Metscape; MGV; Paintomics; Pathos; Pathvisio; ProMetra).

Adapted with modification from [16].

## Supplementary Materials

The following are available online at https://www.mdpi.com/article/10.3390/metabo11110729/s1, Figure S1: title. Overview of the Multi-Omic Characterization of Immune Responses in COVID-19 Patients, Related to Figure S1.
