# Supplementary material for: Single-Cell Multiomics Analysis for Drug Discovery
Source: Metabolites. 2021 Oct 25;11(11):729. doi: 10.3390/metabo11110729 (PMC8623556; doi:10.3390/metabo11110729)
Supplement: Supplementary file 1 [file metabolites-11-00729-s001.zip › metabolites-1416930-supplementary.pdf]

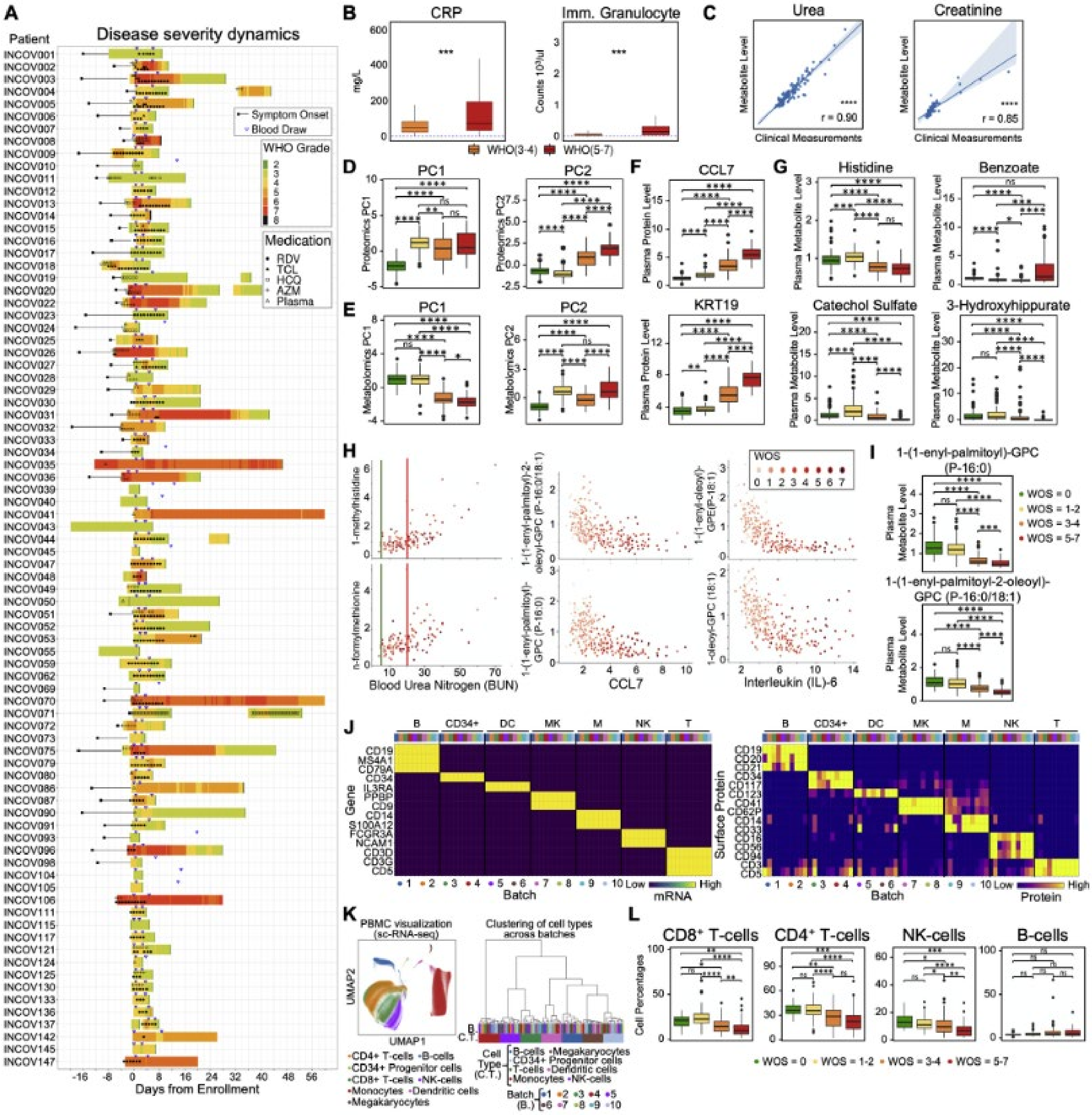

Figure S1. Overview of the Multi-Omic Characterization of Immune Responses in COVID-19 Patients, Related to Figure 1A. The swimmer plot depicting EHR-extracted WOS severity score dynamics for studied patients who were admitted to hospital. Symptom onset and pre-hospitalization (if available) are indicated by black rectangles and lines. WOS is calculated at 6-h intervals during hospitalization. Blood draws are indicated by upside-down blue triangles and administered medications by symbols overlaid on the colored bands. B. Boxplots of clinical data comparing moderate (orange) and severe (red) patient sample values. Ranges that specify normal limits are indicated by the dashedlines. Significance is indicated by: (* $p < 0.05$, ** $p < 0.01$, *** $p < 0.001$, **** $p < 0.0001$). Adapted with permission from [84].
